# Case Report: Surgery combined with targeted therapy for metastatic anorectal malignant mucosal melanoma

**DOI:** 10.3389/fonc.2025.1615122

**Published:** 2025-10-06

**Authors:** Yadong Yao, Pule Liu, Kai Liu, Lirong Lian, Shuo Gao

**Affiliations:** ^1^ Department of General Surgery, The Second Hospital of Lanzhou University, Lanzhou, Gansu, China; ^2^ The Second Clinical Medical College of Lanzhou University, Lanzhou, Gansu, China

**Keywords:** anorectal mucosal melanoma, targeted therapy, KIT mutation, prognosis, surgery

## Abstract

**Background:**

Anorectal mucosal melanoma is a rare tumor with a poor prognosis. Timely diagnosis and treatment are essential for improving patient survival. Currently, there is no standardized treatment approach for this disease. This case report emphasizes the effectiveness of combining surgery with targeted therapy for advanced KIT-mutated anorectal mucosal melanoma, as well as the potential adverse effects during targeted therapy.

**Case report:**

We report a 66-year-old female diagnosed with primary anorectal mucosal melanoma with liver metastasis. She underwent a transanal local excision to remove the primary anorectal mucosal melanoma and ultrasound-guided radiofrequency ablation for the liver metastasis. After surgery, she received targeted therapy with imatinib due to the presence of c-Kit mutations. The results showed the expected clinical efficacy. Unfortunately, the patient later developed portal vein thrombosis and liver dysfunction during the imatinib therapy, necessitating the discontinuation of targeted therapy.

**Conclusions:**

The combination of transanal local excision of the primary lesion and radiofrequency ablation of liver metastases, complemented by imatinib-targeted therapy, represents a feasible therapeutic strategy for advanced KIT-mutated anorectal mucosal melanoma. This strategy may prolong patient survival; however, potential adverse events require careful monitoring.

## Introduction

Anorectal mucosal melanoma (AMM) is a rare and aggressive malignancy, representing only 0.4–1.6% of all melanomas (1). Despite its rarity, the incidence of AMM has risen in recent decades and is higher in women than in men (2, 3). The clinical manifestations of AMM are typically nonspecific, complicating the diagnostic process (4, 5). Consequently, AMM is frequently diagnosed at an advanced stage, often with local-regional or distant metastases (2). Effective treatment options for advanced AMM are currently limited, largely due to insufficient data on prognostic factors and survival outcomes (5, 6). This case report, combined with a literature review, aims to provide a reference for the diagnosis, management, and prognosis of advanced KIT-mutated AMM.

## Case presentation

**Figure 1:**
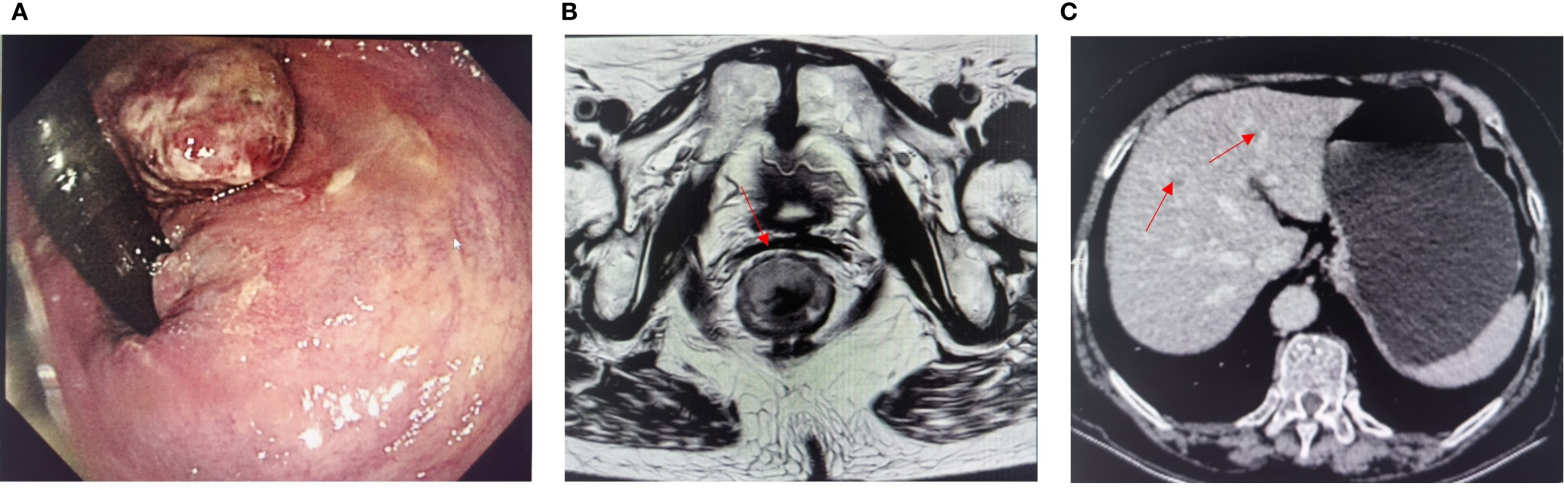
**(A)** Preoperative colonoscopy image. **(B)** Magnetic resonance imaging evaluation before surgery. **(C)** Enhanced CT showed liver metastases (red arrows indicate lesions).

**Figure 2:**
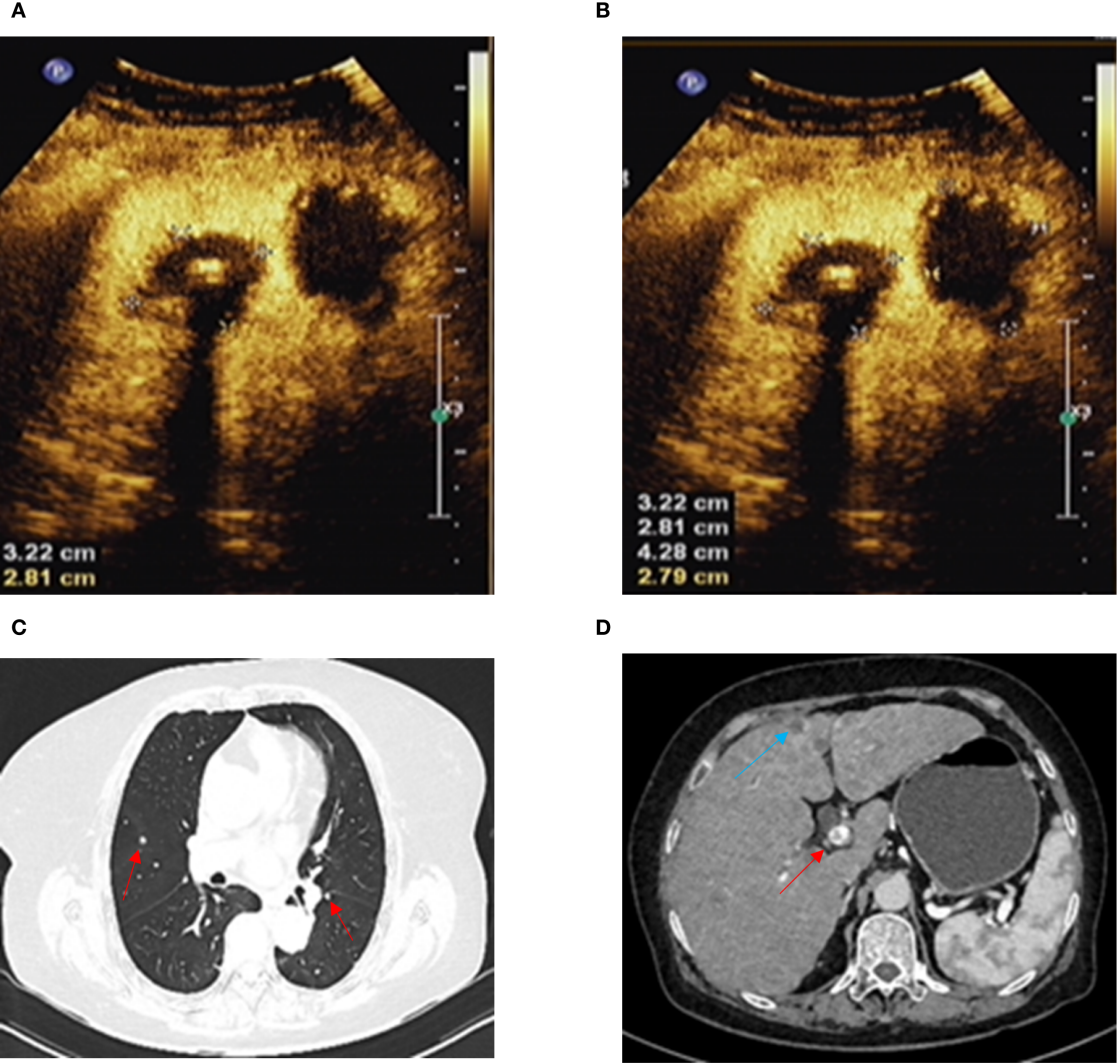
**(A, B)** Ultrasound contrast shows complete inactivation of liver metastases. **(C)** Chest-enhanced CT shows lung metastasis (red arrow). **(D)** Abdominal-enhanced CT shows liver metastasis (blue arrow) and portal vein thrombosis (red arrow).

**Figure 3:**
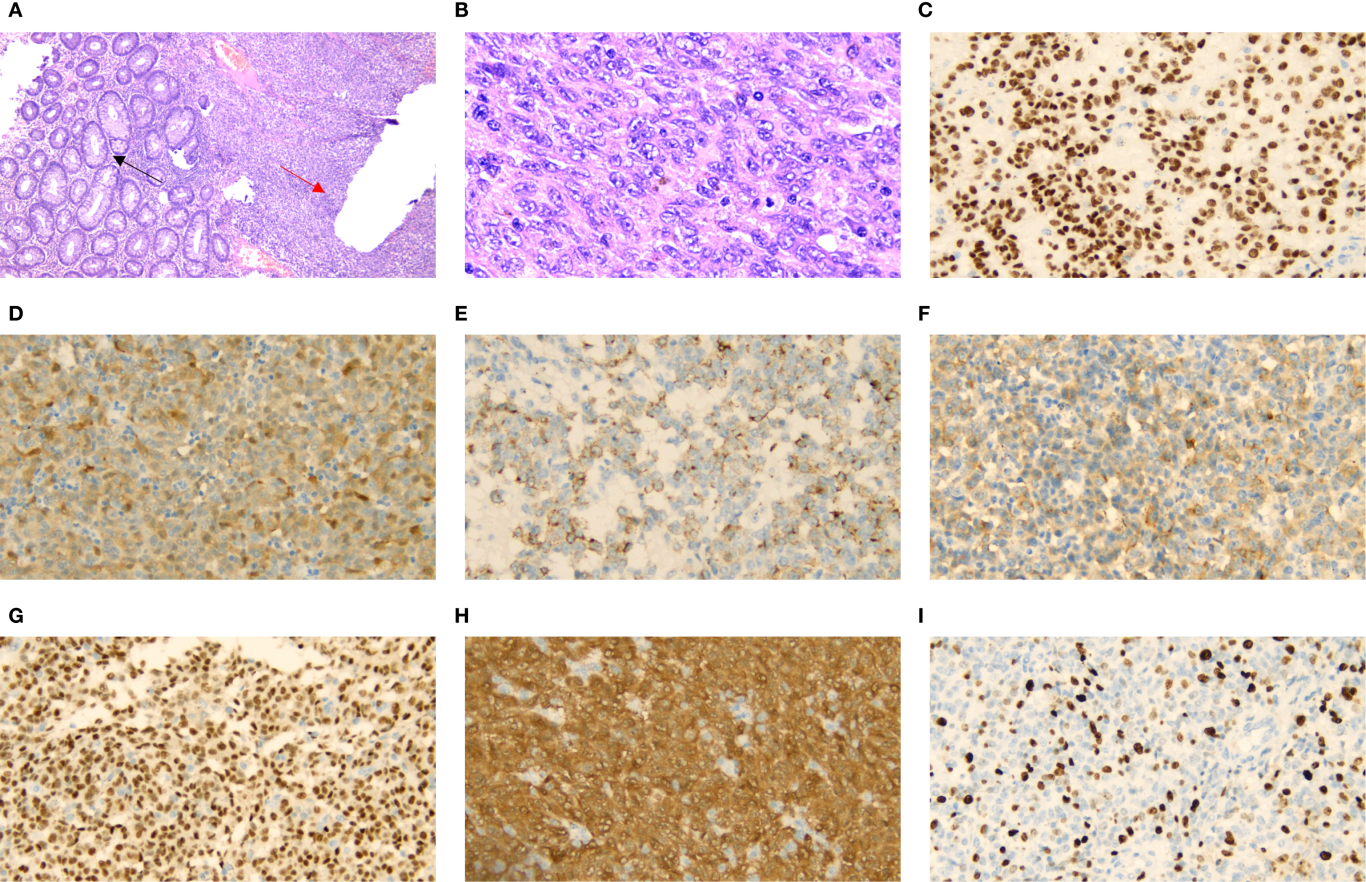
H&E staining showing rectal glandular cells (black arrow) and tumor cells (red arrow) [**(A)** X40]. High-power microscopic examination shows various rectal melanoma cell morphologies [**(B)** X400]. Immunohistochemical staining of, SOX100 [**(C)** X400], S-100 [**(D)** X400], HMB-45 [**(E)** X400], Mela A [**(F)** X400], PRAME [**(G)** X400], p16 [**(H)** X400] and Ki-67[**(I)** X400] were positive.

**Figure 4:**
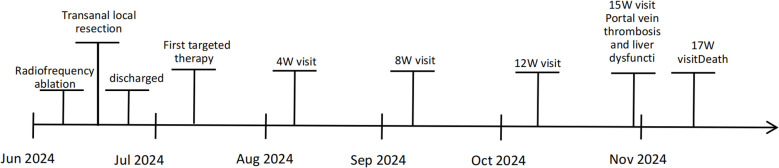
Diagnosis, treatment, and visit timeline for patient.

A 66-year-old woman with a complaint of intermittent anorectal bleeding and pain for 1 month. During this period, she underwent a colonoscopy at a local hospital, which revealed the presence of a rectal tumor. Due to limited medical conditions in local hospitals, surgical treatment was not performed. Subsequently, she presented to our hospital. The patient has no history of harmful habits or significant past medical conditions, and there is no relevant family history of genetic disorders. Physical examination revealed a non-tender mass measuring 4 × 3 centimeters in the anorectal region, located approximately 4 cm from the anal margin. Colonoscopy showed a rectal lesion of about 4 cm × 3 cm × 3 cm, located within 5cm of the anal verge, with visual erosion and hemorrhage (**Figure 1A**). Biopsy was taken for the final diagnosis. Rectal magnetic resonance imaging (MRI) showed a soft tissue mass protruding from the rectal wall, with no local lymph node enlargement (**Figure 1B**). Enhanced computed tomography (CT) of the chest and abdomen detected an occupying lesion protruding from the rectal wall into the intestinal lumen, as well as a circular low-density shadow in the 4/8 liver segment along with mild enhancement. No lymph node enlargement was found in the local area. (**Figure 1C**). Hematological examinations, in review including blood count, biochemical tests, carcinoembryonic antigen, and alpha-fetoprotein levels, were within normal ranges, except that CA 199 was 29.70U/ml. Histological biopsy findings (HE staining) showed that the tumor was composed of spindle-shaped and oval-shaped cells, exhibiting significant cellular atypia and nuclear division. Immunohistochemical staining revealed positive results for HMB 45. Expression of S-100 was negative. The expression level of Ki67 was 40%. Based on the histopathological cell morphology, immunohistochemistry, and imaging examination, the patient was preliminarily diagnosed with a primary AMM with liver metastasis. After obtaining written informed consent. The patient underwent transanal local resection for primary AMM and ultrasound-guided radiofrequency ablation for liver metastases. Postoperative contrast-enhanced ultrasound demonstrated complete inactivation of the liver metastases (**Figures 2A, B**). The postoperative pathological examination results (HE staining) revealed negative margins, and the tumor cells exhibited spindle-shaped, oval-shaped, and pleomorphic types (**Figures 3A, B**). The immunohistochemical analysis showed positive staining for SOX100, S-100, HMB-45, Mela A, PRAME, p16, and ki-67 (+70%) (**Figures 3C-I**). Genetic testing identified a mutation in exon 17 of the KIT gene (D820Y), while both the BRAF and NRAS genes were found to be wild-type. The patient recovered well and was discharged on the sixth day after surgery. Three weeks post-surgery, whole-body bone imaging and enhanced CT scans of the chest and abdomen were performed. The results showed that the patient experienced no complications related to the surgery and no signs of tumor recurrence. Blood cell counts and biochemical tests were normal, with the CA 19–9 level at 31.30 U/ml. Subsequently, targeted therapy with imatinib was initiated at a dosage of 400 mg per day. CT scans of the chest, abdomen, and pelvis were performed every two months. However, after 14 weeks of imatinib treatment, the patient experienced intermittent abdominal pain. The blood routine test was normal. Biochemical tests revealed elevated levels of bilirubin, ALT, and AST, with the AST level exceeding 3-fold the upper limit of the normal reference value. The D-dimer level was measured at 4.04 µg/mL. Tumor markers: CA199 was 210.00U/ml, CA125 was 72.80U/ml. A CT scan revealed a portal vein thrombosis, along with new metastases in the liver and lungs (**Figures 2C, D**). No new lesions were found in the anorectal area. An ultrasound of both lower limb blood vessels showed no abnormalities. The patient discontinued imatinib and started receiving anticoagulant and hepatoprotective medication; however, the symptoms did not improve. As a result, the patient forwent further treatment and was discharged, dying two weeks later (**Figure 4**).

## Discussion

Primary malignant melanoma can develop in various locations, including the esophagus, small intestine, anorectal region, skin, and eyes. However, some gastrointestinal melanomas may arise from metastatic cutaneous melanomas (7). In the present case, no direct evidence of cutaneous melanoma was observed; therefore, it was classified as primary AMM. Unlike cutaneous melanomas, anorectal malignant melanoma (AMM) generally exhibits a poorer prognosis owing to delayed diagnosis and its inherently aggressive behavior (3). The five-year survival rate is approximately 20% for localized disease and 0% for metastatic disease (5). Approximately 20% of patients present with distant metastasis at diagnosis, primarily as a result of delayed detection. The liver, lungs, and pelvis are the most commonly affected sites for metastasis (5, 8), consistent with the findings in this case.

AMM often presents with benign polyp-like changes or hemorrhoid-like dark red alterations (4). These features, along with potential infectious manifestations, may mimic other anorectal conditions, leading to misdiagnosis. In this case, colonoscopy revealed a lesion near the dentate line with ulceration but lacking typical pigmentation. Consequently, other differential diagnoses were considered, including gastrointestinal stromal tumors, neurogenic tumors, and various metastatic diseases. Distinguishing primary AMM from other tumors in this region remains challenging due to the lack of specific imaging characteristics (9, 10). Immunohistochemistry (IHC) is widely regarded as the most common method for diagnosing melanoma. Several key markers reported in the literature, including SOX10, HMB45, Melan-A (11, 12), as well as PRAME, p16, and Ki-67 (13, 14), aid in differentiating AMM from other lesions. The diagnosis of AMM with liver metastasis was confirmed through IHC in this case. Following transanal local excision of the primary lesion and radiofrequency ablation for liver metastases, genetic testing was performed to explore further therapeutic options, revealing a c-KIT mutation. The patient subsequently received targeted therapy with imatinib, which led to prolonged survival. Owing to the rarity and aggressive nature of this disease, coupled with the limited number of affected patients, a standard treatment regimen has not been established. Consequently, this case may serve as a valuable reference for the management of advanced KIT-mutated melanoma patients.

Currently, the American Joint Committee on Cancer (AJCC) guidelines have not established an effective staging system for assessing prognosis and guiding treatment in AMM (15). Surgical resection remains the primary treatment for localized disease (16). Decisions regarding local excision (LE) versus abdominoperineal resection (APR) should be made on a case-by-case basis (17). Both procedures aim to improve survival through R0 resection. A recent systematic review and meta-analysis indicated that LE may reduce postoperative complications and preserve sphincter function, without significant survival disadvantage compared to APR (1). In this case, R0 resection of the primary lesion was successfully achieved via LE. During follow-up, no local recurrence or surgery-related complications were observed. These findings indicate that, provided R0 resection is feasible, LE can achieve local control while preserving sphincter function. However, several studies indicate that patients with advanced AMM may not derive substantial survival benefits from surgical intervention alone (18, 19). Consequently, a multimodal treatment approach incorporating surgery has become the principal strategy for managing advanced AMM.

Activating mutations in BRAF or c-KIT have been identified in malignant melanoma, carrying significant implications for the tumor’s response to anticancer agents targeting these kinases. KIT mutations are more prevalent than NRAS or BRAF mutations, with an observed frequency of approximately 24–33% (20, 21). These mutations occur in various regions, specifically exons 9, 11, 13, 17, and 18 (22). Further studies have demonstrated that c-KIT inhibitors represent promising treatment options for patients with KIT-mutated melanoma, particularly those harboring mutations in exons 11 and 13 (23). However, a recent study indicates no significant difference in antitumor activity among the different exons affected by KIT mutations (24). In the context of targeted therapies for KIT mutations, multiple KIT inhibitors are available besides imatinib. Nevertheless, a meta-analysis indicated that bosutinib, nilotinib, and ponatinib are associated with a higher relative risk of hepatotoxicity compared to imatinib (25). In this case, a c-KIT mutation was identified in exon 17, with no BRAF or NRAS mutations detected. The patient received imatinib, achieving a survival duration of five months (from diagnosis to death). In contrast, patients with AMM and liver metastases who do not receive targeted therapy following surgery typically survive only one to two months (26). Therefore, these findings further confirm that the activating mutation in exon 17 of c-KIT is sensitive to c-KIT inhibitors, and imatinib can prolong survival in advanced AMM patients harboring this mutation. These results provide additional evidence for the management of KIT-mutated AMM patients.

Imatinib may also induce adverse effects during the treatment of solid tumors. Common adverse effects include edema, rash, and fatigue. Other adverse events include vomiting and elevated levels of ALT and AST (27). Portal vein thrombosis is exceedingly rare. Previously, only a single case of splenic vein thrombosis was reported in a patient with a gastric stromal tumor following imatinib administration (28). To our knowledge, this represents the first reported case of portal vein thrombosis in patients with advanced AMM. In this case, the patient had no hematological disorders and exhibited no radiological evidence of vascular compression that could predispose to thrombosis. Furthermore, the patient had no history of thrombosis or known genetic predisposition. Long-term use of tyrosine kinase inhibitors has been reported to induce vascular endothelial damage, potentially leading to venous thrombosis (29). However, the patient had been receiving imatinib for less than four months. Therefore, the portal vein thrombosis may be primarily attributed to paraneoplastic syndrome. Another potential adverse event of imatinib therapy is hepatotoxicity. One study indicates that approximately 1.67% of patients may develop grade 3 or 4 elevations in ALT and AST levels (25). These elevations typically occur within the first two months of treatment initiation and are generally reversible (30). It is not possible to definitively conclude that the liver injury in this patient was solely caused by imatinib, particularly considering the presence of portal vein thrombosis. Notably, these rare adverse events precluded continuation of targeted therapy in this patient. Therefore, this study highlights the potential risk of venous thrombosis in patients with advanced KIT-mutated AMM receiving imatinib. Awareness of this risk is crucial to prevent premature discontinuation of targeted therapy due to potential adverse events.

## Conclusion

This article reports the clinical outcomes of a KIT-mutated AMM patient with concurrent liver metastasis who underwent LE and radiofrequency ablation, in addition to targeted therapy with imatinib. Our findings, supported by a review of relevant literature, suggest that this treatment strategy can effectively control local recurrence, improve quality of life, and prolong survival in patients with advanced KIT mutations. Additionally, we observed that patients with advanced AMM receiving imatinib may experience rare but severe adverse events, including portal vein thrombosis. Given the limited reports on this treatment approach and its associated adverse events, further large-scale studies with long-term follow-up are warranted to validate these findings.

## Data Availability

The original contributions presented in the study are included in the article/Supplementary Material. Further inquiries can be directed to the corresponding author.

## References

[1] Jutten E Kruijff S Francken AB Lutke Holzik MF van Leeuwen BL van Westreenen HL. Surgical treatment of anorectal melanoma: a systematic review and meta-analysis. BJS Open. (2021) 5:zrab107. doi: 10.1093/bjsopen/zrab107, PMID: 34958352 PMC8675246

[2] Paolino G Podo Brunetti A De Rosa C Cantisani C Rongioletti F Carugno A. Anorectal melanoma: systematic review of the current literature of an aggressive type of melanoma. Melanoma Res. (2024) 34:487–96. doi: 10.1097/CMR.0000000000001003, PMID: 39361336 PMC11524631

[3] Kahl AR Gao X Chioreso C Goffredo P Hassan I Charlton ME. Presentation, management, and prognosis of primary gastrointestinal melanoma: A population-based study. J Surg Res. (2021) 260:46–55. doi: 10.1016/j.jss.2020.11.048, PMID: 33316759 PMC7946707

[4] Pintor Tortolero J Durán Martinez M Calleja Lozano R. Anorectal melanoma as an unexpected diagnosis for a pigmented mass resembling a thrombosed hemorrhoid. Clin Gastroenterol Hepatol. (2021) 19:e12–3. doi: 10.1016/j.cgh.2019.11.039, PMID: 31786326

[5] Ottaviano M Giunta EF Marandino L Tortora M Attademo L Bosso D. Anorectal and genital mucosal melanoma: diagnostic challenges, current knowledge and therapeutic opportunities of rare melanomas. Biomedicines. (2022) 10:150. doi: 10.3390/biomedicines10010150, PMID: 35052829 PMC8773579

[6] Bleicher J Cohan JN Huang LC Peche W Pickron TB Scaife CL. Trends in the management of anorectal melanoma: A multi-institutional retrospective study and review of the world literature. World J Gastroenterol. (2021) 27:267–80. doi: 10.3748/wjg.v27.i3.267, PMID: 33519141 PMC7814367

[7] Patel JK Didolkar MS Pickren JW Moore RH. Metastatic pattern of Malignant melanoma. Am J Surg. (1978) 135:807–10. doi: 10.1016/0002-9610(78)90171-x, PMID: 665907

[8] Nam S Kim CW Baek SJ Hur H Min BS Baik SH. The clinical features and optimal treatment of anorectal Malignant melanoma. Ann Surg Treat Res. (2014) 87:113–7. doi: 10.4174/astr.2014.87.3.113, PMID: 25247163 PMC4170582

[9] Smith HG Bagwan I Board RE Capper S Coupland SE Glen J. Ano-uro-genital mucosal melanoma UK national guidelines. Eur J Cancer. (2020) 135:22–30. doi: 10.1016/j.ejca.2020.04.030, PMID: 32531566

[10] Xu Z Zhao K Han L Li P Shi Z Huang X. Combining quantitative and qualitative magnetic resonance imaging features to differentiate anorectal Malignant melanoma from low rectal cancer. Precis Clin Med. (2021) 4:119–28. doi: 10.1093/pcmedi/pbab011, PMID: 35694154 PMC8982618

[11] Charifa A Zhang X. Morphologic and immunohistochemical characteristics of anorectal melanoma. Int J Surg Pathol. (2018) 26:725–9. doi: 10.1177/1066896918773177, PMID: 29759015

[12] Tariq MU Ud Din N Ud Din NF Fatima S Ahmad Z. Malignant melanoma of anorectal region: a clinicopathologic study of 61 cases. Ann Diagn Pathol. (2014) 18:275–81. doi: 10.1016/j.anndiagpath.2014.08.002, PMID: 25163722

[13] O’Connor MK Dai H Fraga GR. PRAME immunohistochemistry for melanoma diagnosis: A STARD-compliant diagnostic accuracy study. J Cutan Pathol. (2022) 49:780–6. doi: 10.1111/cup.14267, PMID: 35672262

[14] Lam GT Prabhakaran S Sorvina A Martini C Ung BS Karageorgos L. Pitfalls in cutaneous melanoma diagnosis and the need for new reliable markers. Mol Diagn Ther. (2023) 27:49–60. doi: 10.1007/s40291-022-00628-9, PMID: 36477449

[15] Nagarajan P Piao J Ning J Noordenbos LE Curry JL Torres-Cabala CA. Prognostic model for patient survival in primary anorectal mucosal melanoma: stage at presentation determines relevance of histopathologic features. Mod Pathol. (2020) 33:496–513. doi: 10.1038/s41379-019-0340-7, PMID: 31383963

[16] Menon H Patel RR Cushman TR Amini A Seyedin SN Adams AC. Management and outcomes of primary anorectal melanoma in the United States. Future Oncol. (2020) 16:329–38. doi: 10.2217/fon-2019-0715, PMID: 32067486

[17] Temperley HC O’Sullivan NJ Keyes A Kavanagh DO Larkin JO Mehigan BJ. Optimal surgical management strategy for treatment of primary anorectal Malignant melanoma-a systematic review and meta analysis. Langenbecks Arch Surg. (2022) 407:3193–200. doi: 10.1007/s00423022-02715-1, PMID: 36331615

[18] Fadel MG Mohamed HS Weir J Hayes AJ Larkin J Smith MJ. Surgical management of primary anorectal melanoma: is less more? J Gastrointest Cancer. (2024) 55:714–22. doi: 10.1007/s12029-023-01009-z, PMID: 38180677 PMC11186905

[19] Kohoutova D Worku D Aziz H Teare J Weir J Larkin J. Malignant melanoma of the gastrointestinal tract: symptoms, diagnosis, and current treatment options. Cells. (2021) 10:327. doi: 10.3390/cells10020327, PMID: 33562484 PMC7915313

[20] Wroblewska JP Dias-Santagata D Ustaszewski A Wu CL Fujimoto M Selim MA. Prognostic roles of BRAF, KIT, NRAS, IGF2R and SF3B1 mutations in mucosal melanomas. Cells. (2021) 10(9):2216. doi: 10.3390/cells10092216, PMID: 34571863 PMC8468625

[21] Li H Yang L Lai Y Wang X Han X Liu S. Genetic alteration of Chinese patients with rectal mucosal melanoma. BMC Cancer. (2021) 21:623. doi: 10.1186/s12885-021-08383-6, PMID: 34044811 PMC8161925

[22] Pham DDM Guhan S Tsao H. KIT and melanoma: biological insights and clinical implications. Yonsei Med J. (2020) 61:562–71. doi: 10.3349/ymj.2020.61.7.562, PMID: 32608199 PMC7329741

[23] Jung S Armstrong E Wei AZ Ye F Lee A Carlino MS. Clinical and genomic correlates of imatinib response in melanomas with KIT alterations. Br J Cancer. (2022) 127:1726–32. doi: 10.1038/s41416-022-01942-z, PMID: 35999272 PMC9596433

[24] Larkin J Marais R Porta N Gonzalez de Castro D Parsons L Messiou C. Nilotinib in KIT-driven advanced melanoma: Results from the phase II single-arm NICAM trial. Cell Rep Med. (2024) 5:101435. doi: 10.1016/j.xcrm.2024.101435, PMID: 38417447 PMC10982988

[25] Wang Z Wang X Wang Z Feng Y Jia Y Jiang L. Comparison of hepatotoxicity associated with new BCR-ABL tyrosine kinase inhibitors vs imatinib among patients with chronic myeloid leukemia: A systematic review and meta-analysis. JAMA Netw Open. (2021) 4:e2120165. doi: 10.1001/jamanetworkopen.2021.20165, PMID: 34292334 PMC8299317

[26] Kolosov A Leskauskaitė J Dulskas A. Primary melanoma of the anorectal region: clinical and histopathological review of 17 cases. A retrospective cohort study. Colorectal Dis. (2021) 23:2706–13. doi: 10.1111/codi.15816, PMID: 34270837

[27] Wei X Mao L Chi Z Sheng X Cui C Kong Y. Efficacy evaluation of imatinib for the treatment of melanoma: evidence from a retrospective study. Oncol Res. (2019) 27:495–501. doi: 10.3727/096504018X15331163433914, PMID: 30075827 PMC7848371

[28] Yilmaz H Demirağ G Yılmaz A. Sigmoid sinus thrombosis followed by splenic infarction due to imatinib therapy in a patient with gastrointestinal stromal tumor; hematological side effects of imatinib. J Pharm Pharm Sci. (2021) 24:148–52. doi: 10.18433/jpps31425, PMID: 33784493

[29] Seki Y Nagano O Koda R Morita S Hasegawa G. Pathological findings suggesting vascular endothelial damage in multiple organs in chronic myelogenous leukemia patients on long-term tyrosine kinase inhibitor therapy. Int J Hematol. (2020) 112:584–91. doi: 10.1007/s12185-020-02913-x, PMID: 32557125

[30] Shah RR Morganroth J Shah DR. Hepatotoxicity of tyrosine kinase inhibitors: clinical and regulatory perspectives. Drug Saf. (2013) 36:491–503. doi: 10.1007/s40264-013-0048-4, PMID: 23620168

